# The Peroxisome Proliferator-Activated Receptor α (PPARα) Agonist Pemafibrate Protects against Diet-Induced Obesity in Mice

**DOI:** 10.3390/ijms19072148

**Published:** 2018-07-23

**Authors:** Masaya Araki, Yoshimi Nakagawa, Asayo Oishi, Song-iee Han, Yunong Wang, Kae Kumagai, Hiroshi Ohno, Yuhei Mizunoe, Hitoshi Iwasaki, Motohiro Sekiya, Takashi Matsuzaka, Hitoshi Shimano

**Affiliations:** 1 Department of Internal Medicine (Endocrinology and Metabolism), Faculty of Medicine, University of Tsukuba, 1-1-1 Tennodai, Tsukuba, Ibaraki 305-8575, Japan; s1721279@s.tsukuba.ac.jp (M.A.); asayo.oishi@gmail.com (A.O.); shan@md.tsukuba.ac.jp (S.-i.H.); gracelynwang@yahoo.co.jp (Y.W.); eak_amos@md.tsukuba.ac.jp (K.K.); s1530432@u.tsukuba.ac.jp (H.O.); ymizunoe@md.tsukuba.ac.jp (Y.M.); iwasaki-tkb@umin.ac.jp (H.I.); msekiya@md.tsukuba.ac.jp (M.S.); t-matsuz@md.tsukuba.ac.jp (T.M.); 2 International Institute for Integrative Sleep Medicine (WPI-IIIS), University of Tsukuba, 1-1-1 Tennodai, Tsukuba, Ibaraki 305-8575, Japan; 3 Life Science Center for Survival Dynamics, Tsukuba Advanced Research Alliance (TARA), University of Tsukuba, 1-1-1 Tennodai, Tsukuba, Ibaraki 305-8577, Japan; 4 Japan Agency for Medical Research and Development–Core Research for Evolutional Science and Technology (AMED-CREST), Chiyoda-ku, Tokyo 100-1004, Japan

**Keywords:** SPPARMα, pemafibrate, PPARα, FGF21, obesity, lipid metabolism

## Abstract

Peroxisome proliferator-activated receptor α (PPARα) is a therapeutic target for hyperlipidemia. Pemafibrate (K-877) is a new selective PPARα modulator activating PPARα transcriptional activity. To determine the effects of pemafibrate on diet-induced obesity, wild-type mice were fed a high-fat diet (HFD) containing pemafibrate for 12 weeks. Like fenofibrate, pemafibrate significantly suppressed HFD-induced body weight gain; decreased plasma glucose, insulin and triglyceride (TG) levels; and increased plasma fibroblast growth factor 21 (FGF21). However, compared to the dose of fenofibrate, a relatively low dose of pemafibrate showed these effects. Pemafibrate activated PPARα transcriptional activity in the liver, increasing both hepatic expression and plasma levels of FGF21. Additionally, pemafibrate increased the expression of genes involved in thermogenesis and fatty acid oxidation, including *Ucp1*, *Cidea* and *Cpt1b* in inguinal adipose tissue (iWAT) and the mitochondrial marker *Elovl3* in brown adipose tissue (BAT). Therefore, pemafibrate activates thermogenesis in iWAT and BAT by increasing plasma levels of FGF21. Additionally, pemafibrate induced the expression of *Atgl* and *Hsl* in epididymal white adipose tissue, leading to the activation of lipolysis. Taken together, pemafibrate suppresses diet-induced obesity in mice and improves their obesity-related metabolic abnormalities. We propose that pemafibrate may be useful for the suppression and improvement of obesity-induced metabolic abnormalities.

## 1. Introduction

Dysregulation of nutrient homeostasis is a common character of metabolic disorders, such as obesity, diabetes, cardiovascular diseases and fatty liver disease. Nutrient homeostasis is tightly maintained via the balance between energy production and energy utilization. The rapid increase in the prevalence of obesity-related metabolic diseases, such as diabetes, hyperlipidemia, hypertension and cancer, is a serious health problem worldwide [1]. Obesity occurs when an individual’s caloric intake exceeds their energy expenditure. In obese individuals, fat accumulates in white adipose tissues (WATs) and in a variety of other tissues. In turn, obesity results in insulin resistance, which leads to obesity-related metabolic disorders.

Peroxisome proliferative-activated receptors (PPARs) include PPARα, PPARβ/δ and PPARγ. Upon ligand binding, PPARs form the complexes with the retinoid X receptor and bind to PPAR response elements (PPREs) in the promoter of these target genes. PPARα is predominantly expressed in the liver and WATs but not in skeletal muscles [2]. PPARα controls fatty acid transport and β-oxidation and improves plasma lipid profiles by decreasing triglyceride (TG) levels and increasing high-density lipoprotein (HDL) cholesterol levels. Thus, PPARα has a crucial role in the regulation of lipid metabolism. Fibrates including gemfibrozil, bezafibrate and fenofibrate are synthetic PPARα agonists that decrease plasma TG levels and increase HDL-cholesterol levels in patients with hyperlipidemia and Type 2 diabetes, thereby preventing coronary heart disease and stroke [3,4,5,6,7]. In addition, treatment with PPARα agonists in animal models of obesity attenuates adiposity and adipocyte hypertrophy and improves glucose metabolism defects, such as hyperglycemia, glucose intolerance and insulin resistance [2,8,9]. However, these drugs are weak agonists of PPARα and have low substrate selectivity, resulting in the need for high doses clinically. Therefore, a more potent and selective PPARα agonist is needed for patients with metabolic syndrome. Pemafibrate (K-877), a novel selective PPARα modulator SPPARMα, increases PPARα transcriptional activity [10,11] and elicits greater PPARα activation than fibrates, with lower EC_50_ values and higher PPAR subtype selectivity [12]. Pemafibrate has an acidic region as fibrates, which contains unique benzoxazole and phenoxyalkyl side-chains, thereby leading to a greater activation of PPARα transcriptional activity and selectivity [13]. Moreover, while other PPARα agonists bind only with one of the Y-shaped ligand-binding pockets of PPARα, pemafibrate binds to the entire cavity region [10]. However, the effects of pemafibrate on obesity and diabetes remain unknown.

The expression of fibroblast growth factor 21 (FGF21), a hormone secreted by the liver, is regulated by PPARα and cAMP-responsive element-binding protein H (CREBH, encoded by *Creb3l3*) during fasting [14,15,16,17]. FGF21 stimulates hepatic ketogenesis and gluconeogenesis to adapt to fasting [18]. FGF21 activates cellular signaling by binding to a cell-surface receptor complex composed of β-Klotho and an FGF receptor 1 (FGFR1) [19,20]. As both β-Klotho and FGFR1 are abundantly expressed in WATs [21], FGF21 regulates the metabolic processes in WATs, including lipogenesis, lipolysis and fatty acid oxidation [22,23]. FGF21 induces the expression of uncoupling protein 1 (*Ucp1*), a thermogenic gene in WATs, activating energy expenditure [22,24]. FGF21 improves energy homeostasis by increasing hepatic fatty acid oxidation and ketogenesis in the liver, thermogenesis of brown adipose tissue (BAT) and browning of WATs, thereby increasing whole-body energy expenditure in mammals. Therefore, FGF21 is a therapeutic target for obesity and obesity-related metabolic diseases [25].

This study investigates the pharmacological effects of pemafibrate versus those of fenofibrate on the progression of obesity in diet-induced obesity (DIO) mice.

## 2. Results

### 2.1. Pemafibrate Suppresses High-Fat Diet (HFD)-Induced Obesity in Mice

**Figure 1:**
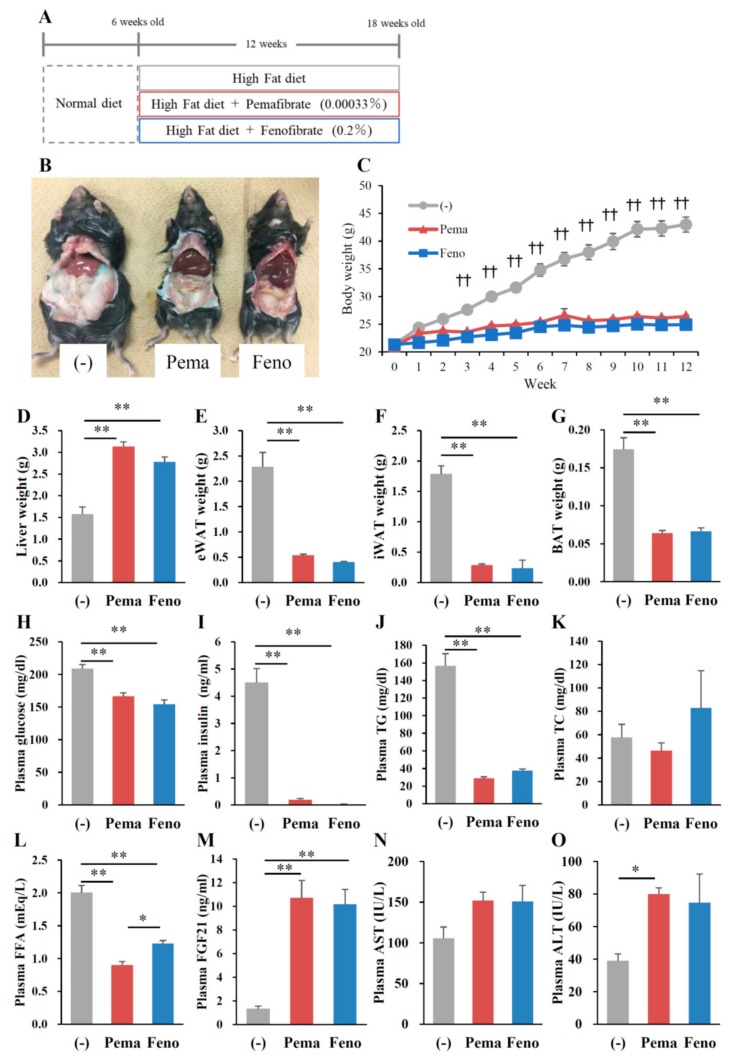
Pemafibrate reduces plasma lipid levels in WT mice fed with high-fat diet (HFD) for 12 weeks. Six-week-old male WT mice were fed HFD plus pemafibrate (0.00033%) or fenofibrate (0.2%) for 12 weeks (**A**); (**B**) Representative pictures of mice; (**C**) body growth curve; †† *p* < 0.01; untreated mice vs. pemafibrate- and fenofibrate-treated mice; (**D**) liver weight; (**E**) eWAT weight; (**F**) iWAT weight; (**G**) BAT weight; and concentrations of (**H**) plasma glucose; (**I**) insulin; (**J**) TG; (**K**) TC; (**L**) FFA; (**M**) FGF21; (**N**) AST and (**O**) ALT. All values are the means ± standard error of the mean (SEM). *n* = 9 per group; * *p* < 0.05; ** *p* < 0.01.

To compare the effects of PPARα agonists on the progression of obesity, 6-week-old male wild type (WT) mice were fed HFD containing 0.00033% pemafibrate or 0.2% fenofibrate for 12 weeks (Figure 1A). The dose of pemafibrate was optimized to achieve similar effects to that of 0.2% fenofibrate (data not shown). After 12 weeks of pemafibrate or fenofibrate administration, all mice exhibited an apparent reduction in diet-induced adiposity (Figure 1B) and a significantly lower BW (Figure 1C) compared to untreated mice, with no change in food intake in all groups. The liver weights of mice administered either agonist were significantly higher than those of untreated mice (Figure 1D). Previous reports show that both agonists increase liver weight accompanied by hepatocyte hypertrophy [26,27]. The weight of epididymal white adipose tissue (eWAT), inguinal WAT (iWAT) and BAT of mice administered either pemafibrate or fenofibrate was significantly lower than that of untreated mice (Figure 1E–G). The plasma glucose, insulin, TG and free fatty acid (FFA) levels of mice treated with either agonist were significantly lower than those of untreated mice (Figure 1H–J,L). Plasma total cholesterol (TC) levels did not differ between groups (Figure 1K). Consistent with a previous report [28], plasma FGF21 levels were significantly higher in both agonist-administered groups than in untreated mice (Figure 1M). There were no differences in plasma aspartate aminotransferase (AST) levels among mouse groups (Figure 1N). Both agonists significantly increased plasma alanine aminotransferase (ALT) levels but there were no changes between both agonists (Figure 1O). Taken together, these results indicate that the toxicities of both agonists in this study were comparable.

**Figure 2:**
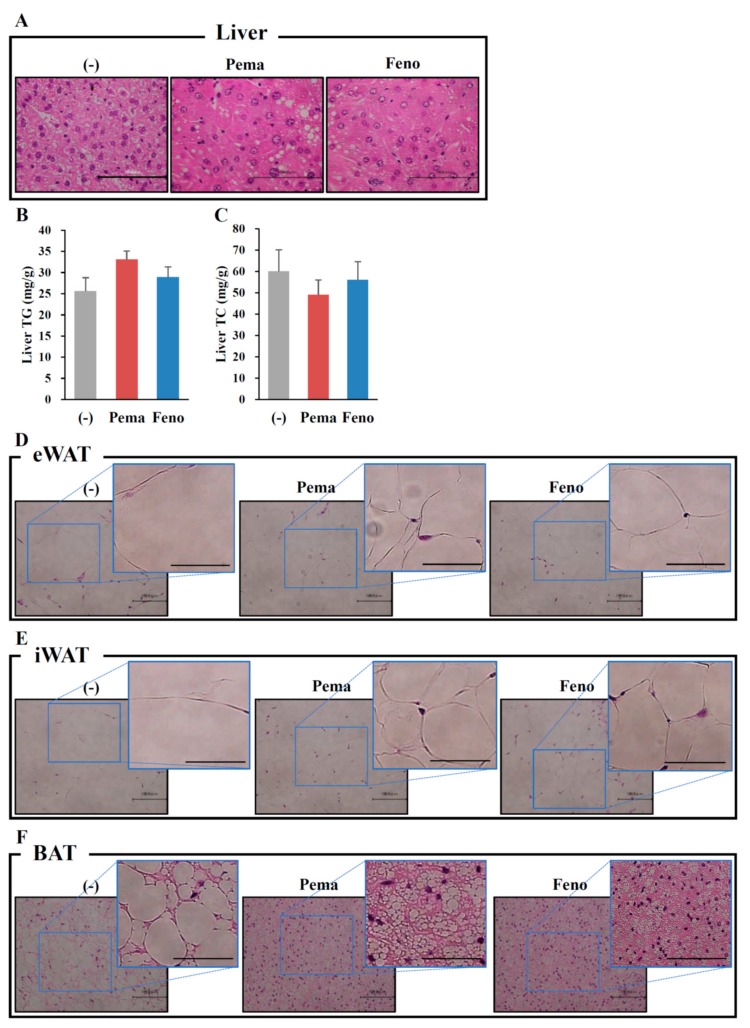
Histological analysis shows that pemafibrate reduces lipid content and cell size in liver and WATs in high-fat diet (HFD)-fed WT mice. Six-week-old male WT mice were fed HFD plus pemafibrate (0.00033%) or fenofibrate (0.2%) for 12 weeks. HE staining analysis in liver (**A**), eWAT (**D**), iWAT (**E**) and BAT (**F**). Liver TG (**B**) and TC (**C**) concentrations; All values are the means ± SEM. *n* = 9 per group; Scale bar: 100 μm.

Morphological analysis with hematoxylin and eosin (HE) staining reveals no apparent differences between the mouse groups (Figure 2A). Consistent with this finding, the quantitative analysis of liver lipids including TG and TC revealed no differences between mouse groups (Figure 2B,C). Surprisingly, HE staining analysis of adipose tissues including iWAT, eWAT and BAT shows that treatment with either agonist decreased the adipocyte size and lipid droplet size compared to those of untreated mice (Figure 2D–F). While BAT adipocytes normally have multilocular lipid droplets, HFD administration changed their appearance to that of WAT adipocytes. Pemafibrate completely suppressed these changes (Figure 2F). These effects of pemafibrate were stronger than those of fenofibrate. These results indicate that both PPARα agonists decrease the cell size in WATs and BAT.

### 2.2. Pemafibrate and Fenofibrate Normalize HFD-Induced Glucose Intolerance and Insulin Resistance

**Figure 3:**
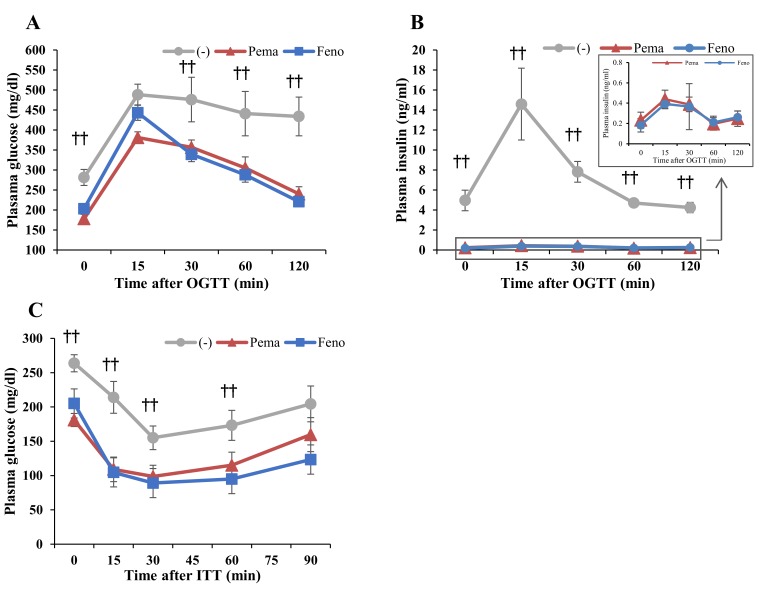
Oral glucose tolerance test (OGTT) and insulin tolerance test (ITT) in high-fat diet (HFD)-fed WT mice treated with pemafibrate. Six-week-old male WT mice were fed HFD plus pemafibrate (0.00033%) or fenofibrate (0.2%) for 10 weeks. (**A**,**B**) Results of OGTT of these mice. Plasma glucose (**A**) and insulin levels (**B**) during OGTT. (**C**) Results of ITT of mice treated with pemafibrate for 11 weeks. Plasma glucose levels during ITT; All values are the means ± SEM. *n* = 9 per group; †† *p* < 0.01; untreated mice vs. pemafibrate- and fenofibrate-treated mice.

Oral glucose tolerance test (OGTT) results indicate that both PPARα agonists improved the glucose response in DIO mice (Figure 3A,B). During OGTT, plasma glucose and insulin levels of mice administered either agonist were significantly lower than those of untreated mice (Figure 3A,B). The lowering effects of the agonists were similar but the glucose in pemafibrate-administered mice were lower than those of fenofibrate-administered mice at 15 min after glucose injection (Figure 3A). Results of the insulin tolerance test (ITT) indicate that the plasma glucose level of mice administered with either agonist was markedly lower than that of untreated mice (Figure 3C). These results indicate that both agonists improve insulin resistance and glucose intolerance in DIO mice.

### 2.3. Pemafibrate and Fenofibrate Activate PPARα-Mediated Gene Expression in the Liver and iWAT but Not eWAT or BAT of DIO Mice

**Figure 4:**
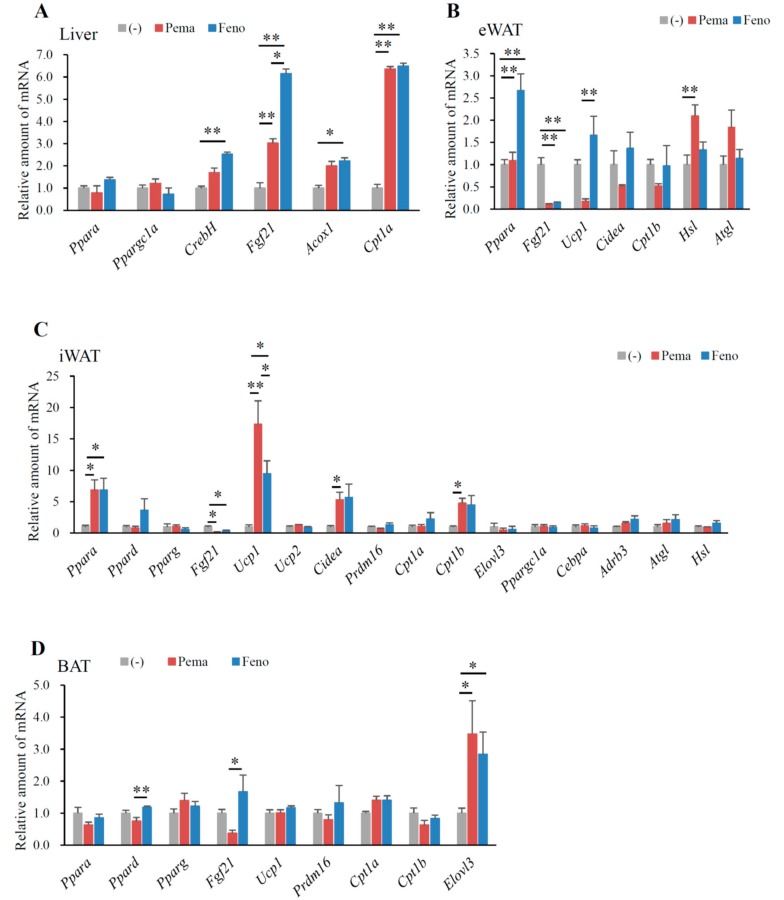
Gene expression in WT mice fed with high-fat diet (HFD) plus pemafibrate for 12 weeks. Six-week-old male WT mice were fed HFD and treated with or without pemafibrate (0.00033%) or fenofibrate (0.2%) for 12 weeks. Gene expression profiles of the liver (**A**), eWAT (**B**), iWAT (**C**) and BAT (**D**); All values are the means ± SEM. *n* = 9 per group; * *p* < 0.05; ** *p* < 0.01.

In mice fed with HFD for 12 weeks, neither agonist changed the expression of *Ppara* and peroxisome proliferative-activated receptor, gamma, coactivator 1 alpha (*Ppargc1a*) in the liver. However, these mice exhibited a significant increase in other PPARα target genes, including *CrebH*, *Fgf21*, acyl-CoA oxidase 1 (*Acox1*) and carnitine palmitoyl transferase 1a (*Cpt1a*) (Figure 4A). Mice treated with fenofibrate exhibited a greater increase in *CrebH* and *Fgf21* expression than did those treated with pemafibrate (Figure 4A). The two agonists equally increased *Acox1* and *Cpt1a* expression (Figure 4A), which are responsible for fatty acid oxidation. These results indicate that pemafibrate acts as a PPARα agonist and activates fatty acid oxidation in the liver of HFD-fed mice.

In eWAT, pemafibrate did not change *Ppara* expression. Pemafibrate decreased the expression of *Fgf21*, *Ucp1*, cell death-inducing DFFA-like effector a (*Cidea*) and carnitine palmitoyl transferase 1b (*Cpt1b*) (Figure 4B). In contrast, pemafibrate increased the expression of adipose triglyceride lipase (*Atgl*) and hormone sensitive lipase (*Hsl*), the rate-limiting enzymes catalyzing triacylglycerol hydrolysis (Figure 4B). Fenofibrate significantly increased *Ppara* and decreased *Fgf21* expression, with no other changes in expression observed when compared to untreated mice (Figure 4B). Pemafibrate decreased *Ucp1* expression (Figure 4B). These findings indicate that pemafibrate does not increase thermogenesis in eWAT. However, pemafibrate significantly increased *Atgl* and *Hsl* expression (Figure 4B), leading to the induction of lipolysis and subsequent reduction in eWAT weight.

In iWAT, both agonists significantly increased the expression of *Ppara* but not *Pparg* (Figure 4C). However, only fenofibrate increased *Ppard* expression (Figure 4C), indicating that fenofibrate has an affinity for PPARδ. Both agonists significantly decreased *Fgf21* expression compared to that of untreated mice (Figure 4C). A previous report indicates that fenofibrate does not change *Fgf21* expression in the iWAT of DIO mice [2]. Moreover, HFD-induced adiposity increased *Fgf21* expression (data not shown); therefore, the suppression of HFD-induced adiposity by either agonist reduced *Fgf21* expression. Importantly, pemafibrate administration resulted in greater increases in *Ucp1* expression than did fenofibrate (Figure 4C). However, either agonist could not change *Ucp2* expression (Figure 4C). Both agonists significantly increased the expression of the browning and mitochondria biogenesis markers *Cidea* and *Cpt1b* (Figure 4C). Taken together, these data indicate that the suppression of BW gain in HFD feeding by pemafibrate contributes to increased thermogenesis. However, PR domain containing 16 (*Prdm16*), a master regulator of the brown/beige program, did not differ between untreated and pemafibrate-treated mice (Figure 4C). In the iWAT of all mice, expressions of adrenergic receptor, beta 3 (*Adrb3*) and the lipolysis genes *Atgl* and *Hsl* were unaltered (Figure 4C).

In BAT, only the expression of *Ppard*, *Fgf21* and ELOVL fatty acid elongase 3 (*Elovl3*), a marker of BAT, were changed in all groups of mice. However, compared to fenofibrate, pemafibrate significantly reduced *Ppard* and *Fgf21* expression in BAT (Figure 4D). Interestingly, the expression of *Elovl3* was significantly elevated in the BAT of mice treated with either agonist when compared to untreated mice (Figure 4D). As Elovl3 is necessary for the synthesis of very long-chain fatty acids as an energy source and full metabolic capacity in BAT [29], both agonists may activate thermogenesis in BAT.

### 2.4. Pemafibrate Ameliorates Obesity-Induced Abnormalities in Obese Mice

**Figure 5:**
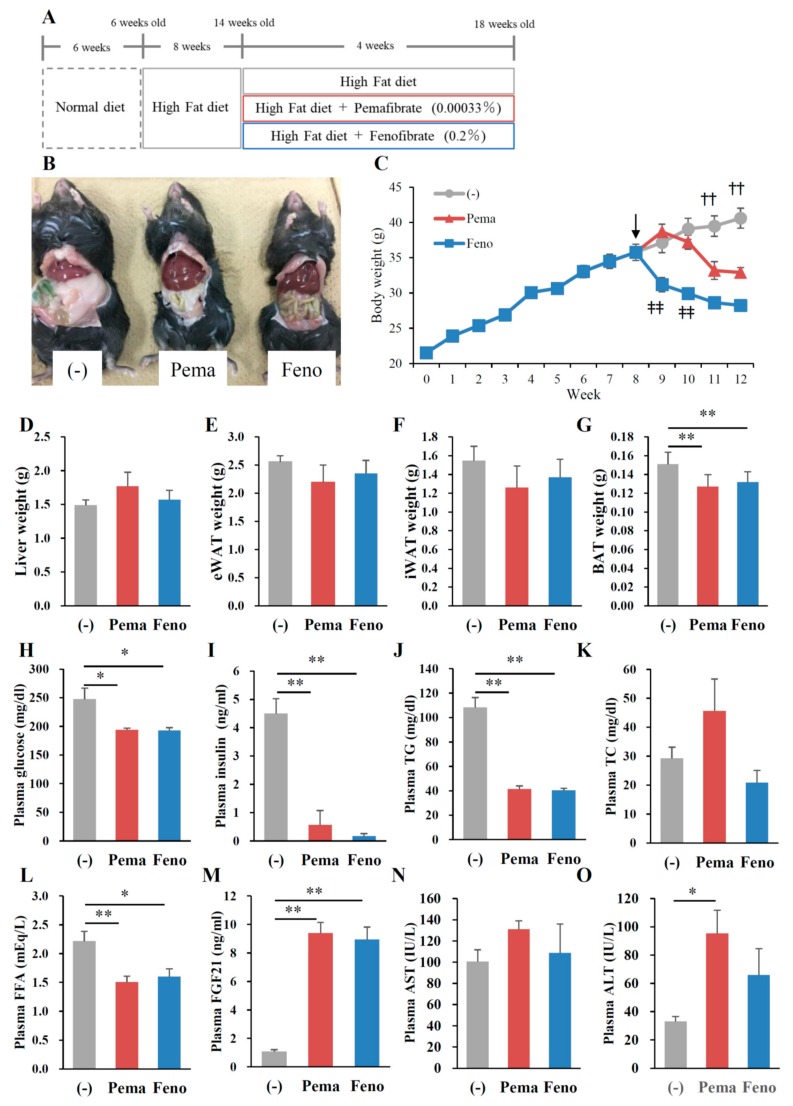
Pemafibrate reduces plasma lipid levels in high-fat diet (HFD)-fed WT mice treated with pemafibrate. Six-week-old male WT mice were fed HFD for 8 weeks and treated with pemafibrate (0.00033%) or fenofibrate (0.2%) for 4 weeks (**A**). (**B**) Representative pictures of mice; (**C**) body growth curve, ↓; start of agonist treatment, ‡‡ *p* < 0.01; untreated mice vs. fenofibrate-treated mice, †† *p* < 0.01; untreated mice vs. pemafibrate- and fenofibrate-treated mice; (**D**) liver weight; (**E**) eWAT weight; (**F**) iWAT weight; (**G**) BAT weight; and concentrations of (**H**) plasma glucose, (**I**) insulin, (**J**) TG, (**K**) TC, (**L**) FFA, (**M**) FGF21, (**N**) AST and (**O**) ALT. All values are the means ± SEM. *n* = 9 per group; * *p* < 0.05; ** *p* < 0.01.

To investigate whether these drugs can improve obesity-related abnormalities in obese mice, mice were fed HFD for 8 weeks and then fed an HFD plus 0.00033% pemafibrate or 0.2% fenofibrate for 4 weeks (Figure 5A). As seen in Figure 1B, both agonists decreased diet-induced adiposity compared with untreated mice (Figure 5B). BW decreases were significant after 3 weeks of pemafibrate and 1 week of fenofibrate administration when compared to untreated mice (Figure 5C). Pemafibrate-treated mice exhibited a trend toward increased liver weight and decreased eWAT and iWAT weights (Figure 5D–F). Compared with untreated mice, fenofibrate showed no difference in liver, eWAT and iWAT weights (Figure 5D–F). Both agonist administration decreased the BAT weight (Figure 5G). Both agonists significantly and similarly reduced plasma glucose, insulin, TG and FFA levels (Figure 5H–J,L). However, only pemafibrate increased plasma TC levels but not significantly (Figure 5K). Both agonists significantly and similarly increased plasma FGF21 levels (Figure 5M). There was difference in plasma AST levels among mouse groups (Figure 5N). Pemafibrate significantly increased plasma ALT levels compared with untreated mice and there were no differences between two agonists (Figure 5O). Taken together, these results indicate that the toxicities of both agonists used in this study were comparable.

**Figure 6:**
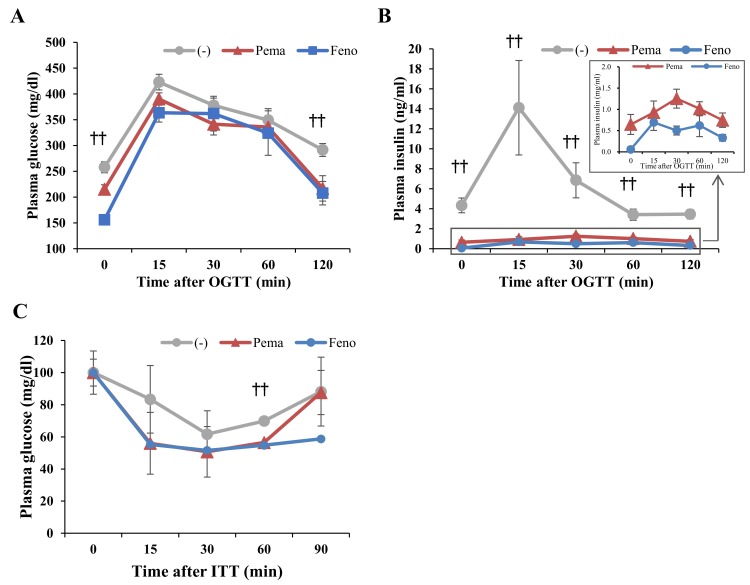
Oral glucose tolerance test (OGTT) and insulin tolerance test (ITT) of high-fat diet (HFD)-induced obese mice treated with pemafibrate. Six-week-old male WT mice were fed HFD for 8 weeks and then treated with pemafibrate (0.00033%) or fenofibrate (0.2%). (**A**,**B**) Results of OGTT of mice treated with pemafibrate or fenofibrate for 2 weeks. Plasma glucose (**A**) and insulin levels (**B**) during OGTT. (**C**) Results of ITT of mice treated with pemafibrate or fenofibrate for 3 weeks. Plasma glucose levels during ITT. All values are the means ± SEM. *n* = 9 per group; †† *p* < 0.01 untreated mice vs. pemafibrate- and fenofibrate-treated mice.

OGTT results show that the plasma glucose levels of mice treated with either agonist were significantly lower than those of untreated mice at 0 and 120 min of testing (Figure 6A). The plasma insulin levels of mice treated with either agonist were significantly lower than those of untreated mice (Figure 6B). ITT results showed no difference in plasma glucose levels between any of the groups of mice; however, at 60 min, mice treated with either agonist exhibited reduced plasma glucose levels compared with untreated mice (Figure 6C).

### 2.5. Pemafibrate Treatment Alters the Expression of Genes Related to Thermogenesis in iWAT and BAT of Mice Fed with Modest Fat (MF) Diet

**Figure 7:**
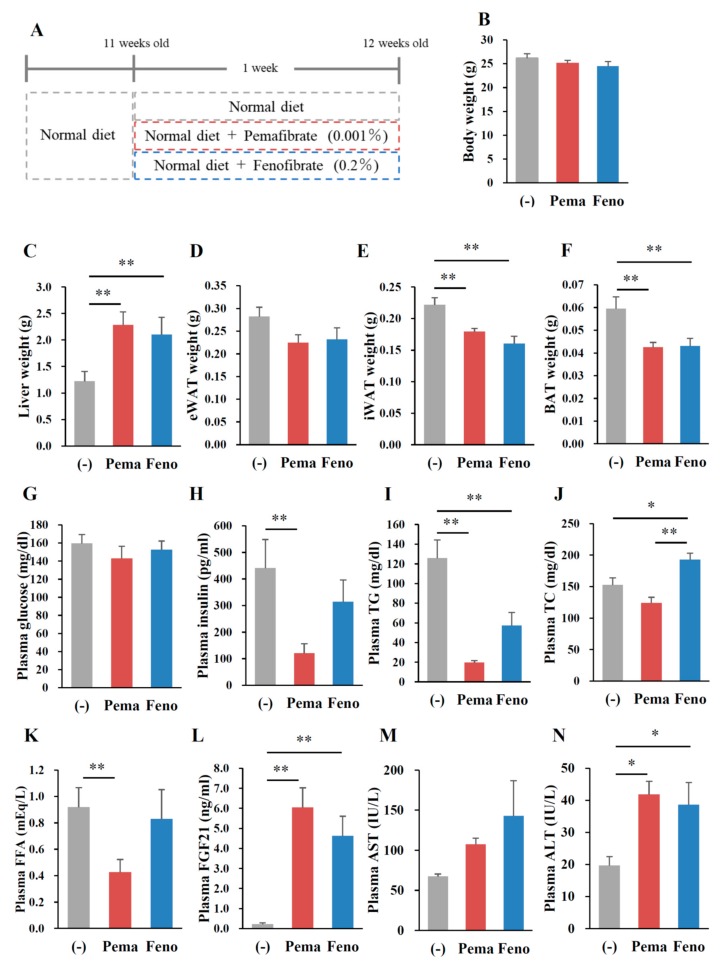
Pemafibrate reduces plasma lipid levels in WT mice fed with (modest-fat) MF diet. Eleven-week-old male WT mice were fed MF diet plus pemafibrate (0.001%) or fenofibrate (0.2%) for 1 week. Body weight (**A**); liver weight (**B**); eWAT weight (**C**); iWAT weight (**D**); BAT weight (**E**); muscle weight (**F**); and concentrations of plasma glucose (**G**), insulin (**H**), TG (**I**), TC (**J**), FFA (**K**), FGF21 (**L**), AST (**M**) and ALT (**N**). All values are the means ± SEM. *n* = 8 per group; * *p* < 0.05; ** *p* < 0.01.

**Figure 8:**
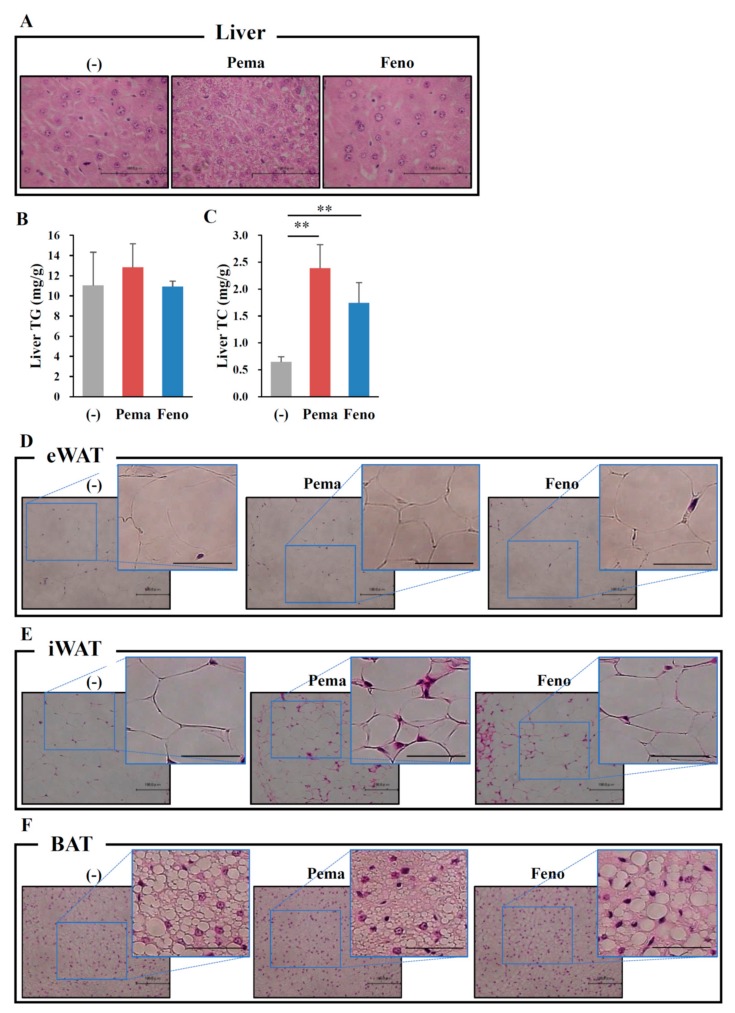
Histological analysis shows that pemafibrate reduces lipid contents and cell size in the liver and WATs in MF-diet fed WT mice. Eleven-week-old male WT mice were fed MF diet plus pemafibrate (0.001%) or fenofibrate (0.1%) for 1 week. HE staining analysis of liver (**A**), eWAT (**D**), iWAT (**E**), BAT (**F**) and concentrations of liver TG (**B**) and TC (**C**). All values are the means ± SEM. *n* = 8 per group; ** *p* < 0.01; *n* = 9–13 per group. Scale bar: 100 μm.

**Figure 9:**
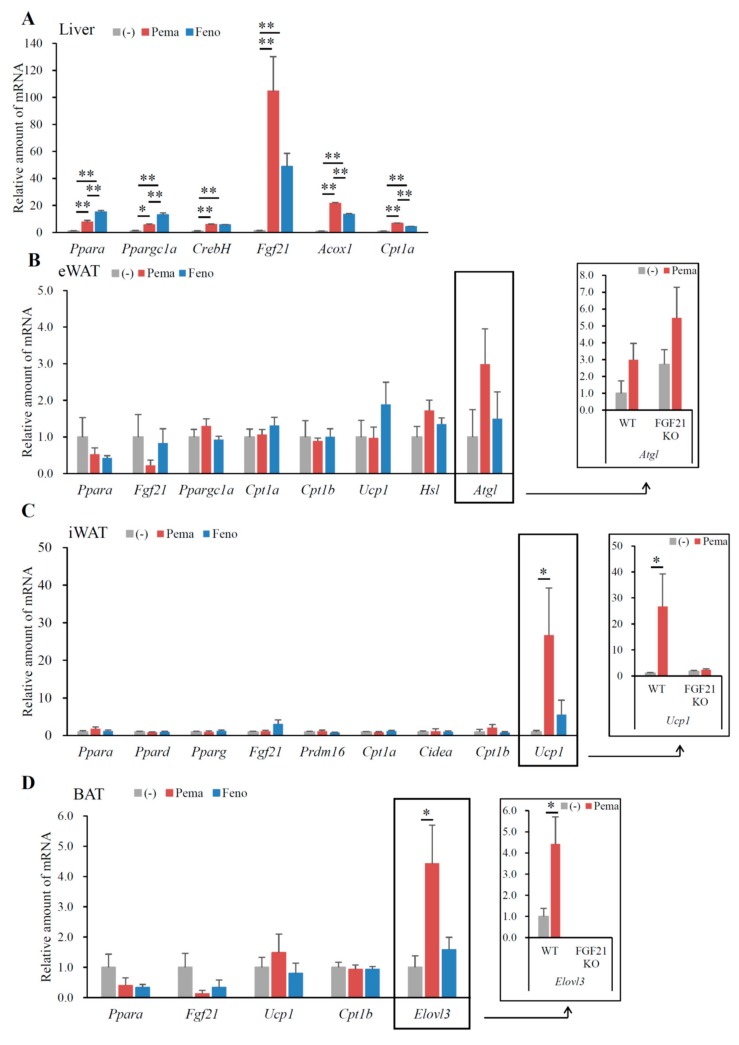
Gene expression in WT mice fed with MF diet plus pemafibrate for 1 week. Eleven-week-old male WT and FGF21 KO mice were fed MF diet plus pemafibrate (0.001%) or fenofibrate (0.1%) for 1 week. Gene expression profiles of the liver (**A**), eWAT (**B**), iWAT (**C**) and BAT (**D**). All values are the means ± SEM. *n* = 8 per group; * *p* < 0.05; ** *p* < 0.01.

Mice fed with MF diet plus 0.001% pemafibrate or 0.2% fenofibrate for 1 week (Figure 7A), as previously optimized [28], exhibited no difference in BW from those of untreated mice (Figure 7B). Mice treated with either pemafibrate or fenofibrate exhibited significantly higher liver weight (Figure 7C), a trend toward lower eWAT (Figure 7D) and significantly lower iWAT and BAT weights (Figure 7E,F) than did untreated mice. While no difference in plasma glucose levels was observed between mouse groups (Figure 7G), pemafibrate significantly decreased plasma insulin levels and fenofibrate treatment trended toward decreased plasma insulin level (Figure 7H). Plasma TG levels in both agonist-administered mice were significantly lower than those of untreated mice, with pemafibrate having greater effects than fenofibrate (Figure 7I). Pemafibrate treatment trended toward decreased plasma TC levels compared with untreated mice but the difference was not significant (Figure 7J). Fenofibrate significantly increased plasma TC levels compared with untreated and pemafibrate-treated mice (Figure 7J). Pemafibrate significantly decreased plasma FFA levels compared with untreated mice (Figure 7K). Both agonists markedly increased plasma FGF21 to the same levels (Figure 7L). There was no difference in plasma AST levels among mouse groups (Figure 7M). Both agonists significantly increased plasma ALT levels compared with untreated mice but there were no differences between the two agonists (Figure 7N). Taken together, these results indicate that the toxicities of both agonists used in this study were comparable. Hepatic morphological analysis with HE staining revealed no apparent differences between mouse groups (Figure 8A). There were no differences in liver TG contents between mouse groups (Figure 8B). However, the liver TC contents in agonist-treated mice were significantly higher than those of untreated mice (Figure 8C). No morphological differences in eWAT, or BAT were observed between any of the groups of mice (Figure 8D,F). In iWAT, both PPARα agonists decreased the size of adipocytes (Figure 8E). Both agonists significantly increased the expression of *Ppara* and its target genes *Ppargc1a*, *CrebH*, *Fgf21*, *Acox1* and *Cpt1a* in the liver (Figure 9A). Pemafibrate treatment resulted in greater increases in the expression of *Fgf21*, *Acox1* and *Cpt1a* than did fenofibrate; in contrast, fenofibrate treatment caused greater increases in *Ppara* and *Ppargc1a* expression (Figure 9A). In eWAT, *Ppara* expression was lower in mice treated with either agonist. Treatment with pemafibrate but not fenofibrate decreased *Fgf21* expression. Fenofibrate treatment increased *Ucp1* expression (Figure 9B). Pemafibrate-treated mice trended toward increased *Atgl* expression in eWAT compared with untreated and fenofibrate-treated mice (Figure 9B). In iWAT, pemafibrate treatment did not alter the expression of any PPAR family molecules (*Ppara*, *Pparg* and *Ppard*), PPAR target genes (*Fgf21*, *Cpt1a* and *Cpt1b*), or the beige genes *Prdm16* and *Cidea* (Figure 9C). Surprisingly, the expression of *Ucp1*, a thermogenic marker, was markedly increased in pemafibrate-treated mice (Figure 9C). In BAT, the expression of *Ppara* and *Fgf21* was decreased and that of *Ucp1* and *Cpt1b* was unchanged in BAT of mice treated with either agonist (Figure 9D). The expression of *Elovl3*, a thermogenic marker gene, was robustly increased in pemafibrate-treated mice (Figure 9D).

### 2.6. Pemafibrate-Induced Gene Expression Partially Depends on FGF21

To determine the role of FGF21 in pemafibrate-induced changes in gene expression, FGF21 knockout (KO) mice were fed MF diet containing 0.001% pemafibrate for 1 week. Pemafibrate increased *Atgl* expression in the eWAT of both WT and FGF21 KO mice (Figure 9B), suggesting that the upregulation of these genes is not dependent on FGF21. Surprisingly, pemafibrate-induced *Ppara* expression in iWAT was not observed in FGF21 KO mice (Figure 9C), indicating that FGF21 regulates *Ppara* expression in iWAT. Moreover, the pemafibrate-induced *Ucp1* expression seen in the iWAT of WT mice was not observed in FGF21 KO mice (Figure 9C). Similarly, the pemafibrate-induced *Elovl3* expression seen in the BAT of WT mice was not observed in FGF21 KO mice (Figure 9D). Taken together, these findings suggested that pemafibrate-induced hepatic FGF21 production enhances FGF21 signaling in WATs and BAT, leading to browning and thermogenesis in these tissues.

## 3. Discussion

Our results show that pemafibrate significantly suppressed HFD-induced body weight gain, decreased plasma glucose, insulin and triglyceride (TG) levels and increased plasma FGF21 expression in DIO mice. Pemafibrate activated PPARα transcriptional activity in the liver, thereby increasing both hepatic expression and plasma levels of FGF21. In addition, pemafibrate increased the expression of genes involved in thermogenesis and fatty acid oxidation in iWAT and BAT, suggesting that pemafibrate activates thermogenesis in iWAT and BAT by increasing plasma levels of FGF21. Additionally, pemafibrate induced the expression of *Atgl* and *Hsl* in eWAT, leading to the activation of lipolysis. Taken together, these findings indicate that pemafibrate suppresses DIO in mice.

PPARα agonists are reported to suppress obesity and obesity-induced abnormalities in glucose metabolism [2,9,30,31,32]. Moreover, PPARα agonist treatment increases circulating levels of FGF21 in rodents as well as humans [33], suggesting that treatment with a PPARα agonist increases FGF21 signaling in peripheral tissues and improves energy homeostasis. Surely, FGF21 treatment reduces body weight and improves glucose metabolism in mouse models of obesity and diabetes [22,23,34,35]. Compared with classical PPARα agonists, pemafibrate is a novel selective PPARα modulator that increases PPARα transcriptional activity with high selectivity and potency [10,11,28].

We observed that both PPARα agonists strongly suppress BW as well as WAT weight gain in DIO mice. A previous report only indicates that pemafibrate attenuates postprandial hypertriglyceridemia by suppressing the postprandial increase in chylomicrons and the accumulation of chylomicron remnants more effectively than fenofibrate in DIO mice [36]. Therefore, the mechanism of suppression of BW by pemafibrate remains unknown.

Pemafibrate clearly suppressed HFD-induced obesity with no difference in food intake between untreated and pemafibrate-treated mice. This result indicates that pemafibrate increases energy expenditure. PPARα is predominantly expressed in the liver, where it plays a crucial role in many physiological functions, such as the maintenance of whole-body fatty acids and TG metabolism. Pemafibrate significantly increased the expression of *Cpt1a* and *Acox1* genes involved in fatty acid oxidation in the liver, suggesting that this increased expression contributes to the activation of hepatic fatty acid oxidation and the subsequent decrease in plasma TG levels. PPARα is also expressed in WAT and several reports show that the direct activation of PPARα in WAT can regulate WAT functions [31,32,37,38,39]. Moreover, PPARα activation in WAT reportedly increases β-oxidation related gene expression, fatty acid oxidation and oxygen consumption rate [31,39]. PPARα agonist treatment reportedly increases the hepatic expression and plasma levels of FGF21, resulting in the activation of FGF21 signaling in WATs [24]. The PPARα agonist–FGF21–WAT interaction may contribute to the anti-obesity effect of PPARα agonists. Clearly, chronic administration of FGF21 in mice increases energy expenditure and causes weight loss [22,23]. Consistent with this possibility, pemafibrate treatment markedly increased hepatic *Fgf21* expression and plasma FGF21 levels, partially explaining the weight loss effect by pemafibrate.

PPARα directly activates *Atgl* and *Hsl* expression in WAT, inducing lipolysis [40]. Plasma FGF21 can cross the blood–brain barrier [41] and activate the central nervous system [42]. Accordingly, FGF21 activates browning in WAT, accompanied by inducing the expression of *Ucp1* and lipolysis genes such as *Atgl* and *Hsl* via the sympathetic nervous system [43]. Lipolysis in WATs is activated by β3 adrenergic receptor signaling, which increases the expression of *Hsl* and *Atgl*. Activation of this pathway decreases the weight and the lipid droplet size of WATs. Although both agonists induced similar plasma levels of FGF21 HFD-fed mice, only pemafibrate activated the expression of these genes in eWATs. FGF21 deficiency did not suppress pemafibrate-induced expression of these genes, indicating that FGF21 is not involved in their expression in eWATs. Therefore, pemafibrate specifically induces *Atgl* and *Hsl* expression in eWAT.

UCP1 generates heat during thermogenesis by uncoupling oxidative phosphorylation [44]. Thermogenesis inversely correlates with body mass index and adiposity [45,46,47], suggesting that the activation of BAT during thermogenesis protects against obesity and obesity-related metabolic disorders. Pemafibrate treatment induced *Ucp1* expression in iWAT but not in BAT. Recent studies have addressed the conversion of white adipocytes to BAT-like white adipocytes expressing *Ucp1* mRNA. UCP1-expressing brown-like adipocytes (called as beige/bright cells) arise in iWAT in response to cold exposure and β-adrenergic receptor agonists [48,49]. These cells have thermogenic capacity and can protect mice against DIO [50,51]. *UCP1* promoter contains a PPRE, the consensus sequence for PPAR family molecules [52], suggesting that pemafibrate activates *Ucp1* expression by activating PPARα transcriptional activity. A recent study reports that FGF21 induces the conversion of WAT adipocytes to BAT-like adipocytes by inducing the expression of thermogenic genes, including *Ucp1*, *Cidea* and *Cpt1b* [24]. Consistent with this observation, we observed that pemafibrate induces the expression of the thermogenic markers *Ucp1*, *Cidea* and *Cpt1b* expression in iWAT, further supporting the premise that pemafibrate increases thermogenesis and converts cells into BAT-like adipocytes. This cellular change partially explains how pemafibrate suppresses HFD-induced adiposity. Because these genes are PPARα targets [53], pemafibrate may directly affect PPARα transcriptional activity in iWAT, leading to increased gene expression. FGF21 acts directly on BAT and iWAT to increase glucose uptake and substrate mobilization [54]. We observed that the pemafibrate-induced *Ucp1* expression seen in the iWAT of WT mice fed with MF diet did not occur in FGF21 KO mice. Therefore, pemafibrate-PPARα-induced FGF21 induces *Ucp1* expression in iWAT.

*Elovl3* is highly increased in BAT in response to cold exposure [55] and Elovl3 KO mice exhibit defects in lipid recruitment to BAT upon cold exposure [56]. *Elovl3* deficiency leads to a shortage in the fuel supply for fatty acid oxidation. Therefore, Elovl3 is used as a marker for the activation of mitochondria function [57]. BAT activation is accompanied by increased expression of genes related to energy expenditure and fatty acid metabolism, including UCP1 and Elovl3. A previous report suggests that the *Elovl3* promoter contains a putative PPRE [58]. Our data reveal that pemafibrate markedly increased *Elovl3* expression in BAT but did not change *Ppara* or *Fgf21* expression, indicating that pemafibrate does not mediate PPARα activation in BAT. In addition, FGF21-deficient mice fed with MF diet were completely suppressed in *Elovl3* expression. Taken together, our data indicate that the pemafibrate-induced expression of *Elovl3* in BAT is mainly regulated by plasma FGF21.

In conclusion, we observed that compared to the dose of fenofibrate, the relatively low dose of pemafibrate ameliorate obesity-induced abnormalities in DIO mice. The thermogenic functions in the iWAT and BAT of mice treated with pemafibrate were increased via increased hepatic FGF21 production and the direct effects of pemafibrate on PPARα in peripheral tissues, leading to the amelioration of obesity-induced dysfunction. Pemafibrate clearly increased *Ucp1* expression in iWAT, activating browning and thermogenesis. Fenofibrate induced not only *Ppara* but also *Ppard*, whereas pemafibrate induced only *Ppara* in iWAT. Consistent with previous our report that pemafibrate binds to the entire cavity region of ligand-binding pocket of PPARα [10], pemafibrate has the selectivity and power for PPARα activity. These findings indicate that pemafibrate is more selective and powerful than fenofibrate for the improvement of obesity-induced abnormalities in DIO mice.

## 4. Materials and Methods

### 4.1. Reagent

Pemafibrate was kindly provided by Kowa Co. Ltd. (Nagoya, Japan).

### 4.2. Animals

Male C57BL/6J (wild-type, WT) mice were obtained from CLEA Japan. FGF21 knockout (KO) mice were kindly provided by Nobuyuki Itoh and Morichika Konishi at Kyoto University. Six-week-old male WT mice were fed MF diet plus 0.001% pemafibrate or 0.2% fenofibrate for 1 week. Eight-week-old male WT mice were fed HFD; D12492, Research Diet) plus 0.00033% pemafibrate or 0.2% fenofibrate for 12 weeks. Six-week-old WT mice were fed HFD for 8 weeks and then fed HFD plus 0.00033% pemafibrate or 0.2% fenofibrate for 4 weeks. For the OGTT, mice were fasted for 6 h and then orally administered glucose (2 g/kg body weight) after 10 weeks on the HFD. For the ITT, mice were fasted for 4 h and then injected intraperitoneally with regular insulin (Eli Lily) (0.5 U/kg body weight) after 11 weeks on the HFD. All animal husbandry procedures and experiments (17-225) were in compliance with the University of Tsukuba’s Regulations for Animal Experiments and were approved by the Animal Experiment Committee at the University of Tsukuba on 1 June 2017.

### 4.3. Metabolic Measurements

Plasma levels of glucose, insulin, TG, FFA, TC, FGF21, ALT and AST and liver TG and TC levels were measured as described previously [59].

### 4.4. Histological Analysis

Livers, WATs (iWAT and eWAT) and BATs were fixed; embedded in paraffin; sectioned; and stained with HE, as described previously [16].

### 4.5. Analysis of Gene Expression

Total RNA from cells and tissues was prepared using Sepasol (Nacalai, Kyoto, Japan). Before real-time polymerase chain reaction (PCR) analyses, total RNA was reverse transcribed into cDNA using reverse transcriptase according to the manufacturer’s instructions (Takara Bio, Kusatsu, Japan). Real-time PCR was performed using the ABI Prism 7300 system (ABI, Foster City, CA, USA) and the Thermal Cycler Dice Real Time System II (Takara Bio, Kusatsu, Japan) with TB Green Premix Ex Taq II (Takara Bio). Primer sequences are described in Table 1.

### 4.6. Statistical Analyses

Treatment groups were compared using the Tukey–Kramer post-hoc test and differences were considered significant for *p* < 0.05. All data are expressed as mean ± standard error of the mean.

## Figures and Tables

**Table 1:** List of QPCR primer sequences.

*Gene*	Forward Primer	Reverse Primer
*Acox1*	CGATCCAGACTTCCAACATGAG	CCATGGTGGCACTCTTCTTAACA
*Adrb3*	ACAGGAATGCCACTCCAATC	TTAGCCACAACGAACACTCG
*Atgl*	GGATGGCGGCATTTCA	CAAAGGGTTGGGTTGG
*Cebpa*	GCGCAAGAGCCGAGATAAAG	CGGTCATTGTCACTGGTCAACT
*Cidea*	CATCCCCCAAGCCTAG	CTCTGTAGCTGTGCCC
*Cpt1a*	CCTGGGCATGATTGCAAAG	GGACGCCACTCACGATGTT
*Cpt1b*	GGCTGCCGTGGGACATT	TGCCTTGGCTACTTGGTACGA
*CrebH*	CCTGTTTGATCGGCAGGAC	CGGGGGACGATAATGGAGA
*Cyclophilin*	TGGCTCACAGTTCTTCATAACCA	ATGACATCCTTCAGTGGCTTGTC
*Elovl3*	CGTAGTCAGATTCTGG	CCAGAAGAAGTGTTCC
*Fgf21*	AGATCAGGGAGGATGGAACA	TCAAAGTGAGGCGATCCATA
*Hsl*	GAGCGCTGGAGGAGTGTTTT	TGATGCAGAGATTCCCACCTG
*Ppara*	ACGCGAGTTCCTTAAGAACCTG	GTGTCATCTGGATGGTTGCTCT
*Ppard*	TTCCACTATGGAGTTCATGCTTG	TCCGGCAGTTAAGATCACACCT
*Pparg*	TCAACATGGAATGTCGGGTG	ATACTCGAGCTTCATGCGGATT
*Ppargc1a*	TTCAAGATCCTGTTACTACT	ACCTTGAACGTGATCTCACA
*Prdm16*	GGCGAGGAAGCTAGCC	GGTCTCCTCCTCGGCA
*Ucp1*	AGGATGGTGAACCCGACAAC	TTGGATCTGAAGGCGGACTT
*Ucp2*	GACCTCATCAAAGATACTCTCCTGAA	ATCTCGTCTTGACCACATCAACAG

## Abbreviations

Acox1	acyl-CoA oxidase 1
Adrb3	adrenergic receptor, beta 3
ALT	alanine aminotransferase
AST	aspartate aminotransferase
Atgl	adipose triglyceride lipase
BAT	brown adipose tissue
Cidea	cell death-inducing DFFA-like effector a
Cpt1a	carnitine palmitoyl transferase 1a
Cpt1b	carnitine palmitoyl transferase 1b
CREBH	cAMP-responsive element-binding protein H
DIO	diet-induced obesity
Elovl3	ELOVL fatty acid elongase 3
eWAT	epididymal white adipose tissue
FFA	Free fatty acid
FGF21	fibroblast growth factor 21
FGFR1	FGF receptor 1
OGTT	oral glucose tolerance test
HDL	high-density lipoprotein
HE	hematoxylin and eosin
HFD	high-fat diet
Hsl	hormone sensitive lipase
ITT	insulin tolerance test
iWAT	inguinal adipose tissue
KO	knockout
MF	modest fat
Ppargc1a	peroxisome proliferative-activated receptor, gamma, coactivator 1 alpha
PPARα	peroxisome proliferator-activated receptor α
PPRE	PPAR response element
Prdm16	PR domain containing 16
TC	total cholesterol
TG	triglyceride
UCP1	uncoupling protein 1
WT	wild type

## References

[1] Roberto C.A., Swinburn B., Hawkes C., Huang T.T., Costa S.A., Ashe M., Zwicker L., Cawley J.H., Brownell K.D. (2015). Patchy progress on obesity prevention: Emerging examples, entrenched barriers and new thinking. Lancet.

[2] Goto T., Hirata M., Aoki Y., Iwase M., Takahashi H., Kim M., Li Y., Jheng H.F., Nomura W., Takahashi N. (2017). The hepatokine FGF21 is crucial for peroxisome proliferator-activated receptor-α agonist-induced amelioration of metabolic disorders in obese mice. J. Biol. Chem..

[3] Diabetes Atherosclerosis Intervention Study Investigators (2001). Effect of fenofibrate on progression of coronary-artery disease in type 2 diabetes: The Diabetes Atherosclerosis Intervention Study, a randomised study. Lancet.

[4] Bloomfield Rubins H., Davenport J., Babikian V., Brass L.M., Collins D., Wexler L., Wagner S., Papademetriou V., Rutan G., Robins S.J. (2001). Reduction in stroke with gemfibrozil in men with coronary heart disease and low HDL cholesterol: The Veterans Affairs HDL Intervention Trial (VA-HIT). Circulation.

[5] Rubins H.B., Robins S.J., Collins D., Fye C.L., Anderson J.W., Elam M.B., Faas F.H., Linares E., Schaefer E.J., Schectman G. (1999). Gemfibrozil for the secondary prevention of coronary heart disease in men with low levels of high-density lipoprotein cholesterol. Veterans Affairs High-Density Lipoprotein Cholesterol Intervention Trial Study Group. N. Engl. J. Med..

[6] Tanne D., Koren-Morag N., Graff E., Goldbourt U. (2001). Blood lipids and first-ever ischemic stroke/transient ischemic attack in the Bezafibrate Infarction Prevention (BIP) Registry: High triglycerides constitute an independent risk factor. Circulation.

[7] BIP Study Group (2000). Secondary prevention by raising HDL cholesterol and reducing triglycerides in patients with coronary artery disease. Circulation.

[8] Rachid T.L., Penna-de-Carvalho A., Bringhenti I., Aguila M.B., Mandarim-de-Lacerda C.A., Souza-Mello V. (2015). Fenofibrate (PPARα agonist) induces beige cell formation in subcutaneous white adipose tissue from diet-induced male obese mice. Mol. Cell. Endocrinol..

[9] Guerre-Millo M., Gervois P., Raspe E., Madsen L., Poulain P., Derudas B., Herbert J.M., Winegar D.A., Willson T.M., Fruchart J.C. (2000). Peroxisome proliferator-activated receptor α activators improve insulin sensitivity and reduce adiposity. J. Biol. Chem..

[10] Yamamoto Y., Takei K., Arulmozhiraja S., Sladek V., Matsuo N., Han S.I., Matsuzaka T., Sekiya M., Tokiwa T., Shoji M. (2018). Molecular association model of PPARα and its new specific and efficient ligand, pemafibrate: Structural basis for SPPARMα. Biochem. Biophys. Res. Commun..

[11] Raza-Iqbal S., Tanaka T., Anai M., Inagaki T., Matsumura Y., Ikeda K., Taguchi A., Gonzalez F.J., Sakai J., Kodama T. (2015). Transcriptome Analysis of K-877 (a novel selective PPARα modulator (SPPARMα))-regulated genes in primary human hepatocytes and the mouse liver. J. Atheroscler. Thromb..

[12] Fruchart J.C. (2013). Selective peroxisome proliferator-activated receptor α modulators (SPPARMalpha): The next generation of peroxisome proliferator-activated receptor alpha-agonists. Cardiovasc. Diabetol..

[13] Yamazaki Y., Abe K., Toma T., Nishikawa M., Ozawa H., Okuda A., Araki T., Oda S., Inoue K., Shibuya K. (2007). Design and synthesis of highly potent and selective human peroxisome proliferator-activated receptor alpha agonists. Bioorg. Med. Chem. Lett..

[14] Inagaki T., Dutchak P., Zhao G., Ding X., Gautron L., Parameswara V., Li Y., Goetz R., Mohammadi M., Esser V. (2007). Endocrine regulation of the fasting response by PPARalpha-mediated induction of fibroblast growth factor 21. Cell Metab..

[15] Badman M.K., Pissios P., Kennedy A.R., Koukos G., Flier J.S., Maratos-Flier E. (2007). Hepatic fibroblast growth factor 21 is regulated by PPARα and is a key mediator of hepatic lipid metabolism in ketotic states. Cell Metab..

[16] Nakagawa Y., Satoh A., Yabe S., Furusawa M., Tokushige N., Tezuka H., Mikami M., Iwata W., Shingyouchi A., Matsuzaka T. (2014). Hepatic CREB3L3 Controls Whole-Body Energy Homeostasis and Improves Obesity and Diabetes. Endocrinology.

[17] Nakagawa Y., Satoh A., Tezuka H., Han S.I., Takei K., Iwasaki H., Yatoh S., Yahagi N., Suzuki H., Iwasaki Y. (2016). CREB3L3 controls fatty acid oxidation and ketogenesis in synergy with PPARα. Sci. Rep..

[18] Potthoff M.J., Inagaki T., Satapati S., Ding X., He T., Goetz R., Mohammadi M., Finck B.N., Mangelsdorf D.J., Kliewer S.A. (2009). FGF21 induces PGC-1α and regulates carbohydrate and fatty acid metabolism during the adaptive starvation response. Proc. Natl. Acad. Sci. USA.

[19] Ogawa Y., Kurosu H., Yamamoto M., Nandi A., Rosenblatt K.P., Goetz R., Eliseenkova A.V., Mohammadi M., Kuro-o M. (2007). BetaKlotho is required for metabolic activity of fibroblast growth factor 21. Proc. Natl. Acad. Sci. USA.

[20] Suzuki M., Uehara Y., Motomura-Matsuzaka K., Oki J., Koyama Y., Kimura M., Asada M., Komi-Kuramochi A., Oka S., Imamura T. (2008). betaKlotho is required for fibroblast growth factor (FGF) 21 signaling through FGF receptor (FGFR) 1c and FGFR3c. Mol. Endocrinol..

[21] Kurosu H., Choi M., Ogawa Y., Dickson A.S., Goetz R., Eliseenkova A.V., Mohammadi M., Rosenblatt K.P., Kliewer S.A., Kuro-o M. (2007). Tissue-specific expression of betaKlotho and fibroblast growth factor (FGF) receptor isoforms determines metabolic activity of FGF19 and FGF21. J. Biol. Chem..

[22] Coskun T., Bina H.A., Schneider M.A., Dunbar J.D., Hu C.C., Chen Y., Moller D.E., Kharitonenkov A. (2008). Fibroblast growth factor 21 corrects obesity in mice. Endocrinology.

[23] Xu J., Lloyd D.J., Hale C., Stanislaus S., Chen M., Sivits G., Vonderfecht S., Hecht R., Li Y.S., Lindberg R.A. (2009). Fibroblast growth factor 21 reverses hepatic steatosis, increases energy expenditure and improves insulin sensitivity in diet-induced obese mice. Diabetes.

[24] Fisher F.M., Kleiner S., Douris N., Fox E.C., Mepani R.J., Verdeguer F., Wu J., Kharitonenkov A., Flier J.S., Maratos-Flier E. (2012). FGF21 regulates PGC-1α and browning of white adipose tissues in adaptive thermogenesis. Genes Dev..

[25] Gaich G., Chien J.Y., Fu H., Glass L.C., Deeg M.A., Holland W.L., Kharitonenkov A., Bumol T., Schilske H.K., Moller D.E. (2013). The effects of LY2405319, an FGF21 analog, in obese human subjects with type 2 diabetes. Cell Metab..

[26] Miura Y., Hosono M., Oyamada C., Odai H., Oikawa S., Kondo K. (2005). Dietary isohumulones, the bitter components of beer, raise plasma HDL-cholesterol levels and reduce liver cholesterol and triacylglycerol contents similar to PPARα activations in C57BL/6 mice. Br. J. Nutr..

[27] Chan S.M., Zeng X.Y., Sun R.Q., Jo E., Zhou X., Wang H., Li S., Xu A., Watt M.J., Ye J.M. (2015). Fenofibrate insulates diacylglycerol in lipid droplet/ER and preserves insulin signaling transduction in the liver of high fat fed mice. Biochim. Biophys. Acta.

[28] Takei K., Han S.I., Murayama Y., Satoh A., Oikawa F., Ohno H., Osaki Y., Matsuzaka T., Sekiya M., Iwasaki H. (2017). The selective PPARα modulator K-877 efficiently activates the PPARα pathway and improves lipid metabolism in mice. J. Diabetes Investig..

[29] Ediger B.N., Lim H.W., Juliana C., Groff D.N., Williams L.T., Dominguez G., Liu J.H., Taylor B.L., Walp E.R., Kameswaran V. (2017). LIM domain-binding 1 maintains the terminally differentiated state of pancreatic beta cells. J. Clin. Investig..

[30] Takahashi H., Goto T., Yamazaki Y., Kamakari K., Hirata M., Suzuki H., Shibata D., Nakata R., Inoue H., Takahashi N. (2015). Metabolomics reveal 1-palmitoyl lysophosphatidylcholine production by peroxisome proliferator-activated receptor α. J. Lipid Res..

[31] Goto T., Lee J.Y., Teraminami A., Kim Y.I., Hirai S., Uemura T., Inoue H., Takahashi N., Kawada T. (2011). Activation of peroxisome proliferator-activated receptor-α stimulates both differentiation and fatty acid oxidation in adipocytes. J. Lipid Res..

[32] Tsuchida A., Yamauchi T., Takekawa S., Hada Y., Ito Y., Maki T., Kadowaki T. (2005). Peroxisome proliferator-activated receptor (PPAR) α activation increases adiponectin receptors and reduces obesity-related inflammation in adipose tissue: Comparison of activation of PPARα, PPARγ and their combination. Diabetes.

[33] Galman C., Lundasen T., Kharitonenkov A., Bina H.A., Eriksson M., Hafstrom I., Dahlin M., Amark P., Angelin B., Rudling M. (2008). The circulating metabolic regulator FGF21 is induced by prolonged fasting and PPARα activation in man. Cell Metab..

[34] Kharitonenkov A., Shiyanova T.L., Koester A., Ford A.M., Micanovic R., Galbreath E.J., Sandusky G.E., Hammond L.J., Moyers J.S., Owens R.A. (2005). FGF-21 as a novel metabolic regulator. J. Clin. Investig..

[35] Xu J., Stanislaus S., Chinookoswong N., Lau Y.Y., Hager T., Patel J., Ge H., Weiszmann J., Lu S.C., Graham M. (2009). Acute glucose-lowering and insulin-sensitizing action of FGF21 in insulin-resistant mouse models—Association with liver and adipose tissue effects. Am. J. Physiol. Endocrinol. Metab..

[36] Sairyo M., Kobayashi T., Masuda D., Kanno K., Zhu Y., Okada T., Koseki M., Ohama T., Nishida M., Sakata Y. (2018). A Novel Selective PPARα Modulator (SPPARMα), K-877 (Pemafibrate), Attenuates Postprandial Hypertriglyceridemia in Mice. J. Atheroscler. Thromb..

[37] Hiuge A., Tenenbaum A., Maeda N., Benderly M., Kumada M., Fisman E.Z., Tanne D., Matas Z., Hibuse T., Fujita K. (2007). Effects of peroxisome proliferator-activated receptor ligands, bezafibrate and fenofibrate, on adiponectin level. Arterioscler. Thromb. Vasc. Biol..

[38] Mazzucotelli A., Viguerie N., Tiraby C., Annicotte J.S., Mairal A., Klimcakova E., Lepin E., Delmar P., Dejean S., Tavernier G. (2007). The transcriptional coactivator peroxisome proliferator activated receptor (PPAR)γ coactivator-1 α and the nuclear receptor PPAR α control the expression of glycerol kinase and metabolism genes independently of PPAR γ activation in human white adipocytes. Diabetes.

[39] Ribet C., Montastier E., Valle C., Bezaire V., Mazzucotelli A., Mairal A., Viguerie N., Langin D. (2010). Peroxisome proliferator-activated receptor-α control of lipid and glucose metabolism in human white adipocytes. Endocrinology.

[40] Bolsoni-Lopes A., Festuccia W.T., Farias T.S., Chimin P., Torres-Leal F.L., Derogis P.B., de Andrade P.B., Miyamoto S., Lima F.B., Curi R. (2013). Palmitoleic acid (n-7) increases white adipocyte lipolysis and lipase content in a PPARα-dependent manner. Am. J. Physiol. Endocrinol. Metab..

[41] Hsuchou H., Pan W., Kastin A.J. (2007). The fasting polypeptide FGF21 can enter brain from blood. Peptides.

[42] Fon Tacer K., Bookout A.L., Ding X., Kurosu H., John G.B., Wang L., Goetz R., Mohammadi M., Kuro-o M., Mangelsdorf D.J. (2010). Research resource: Comprehensive expression atlas of the fibroblast growth factor system in adult mouse. Mol. Endocrinol..

[43] Douris N., Stevanovic D.M., Fisher F.M., Cisu T.I., Chee M.J., Nguyen N.L., Zarebidaki E., Adams A.C., Kharitonenkov A., Flier J.S. (2015). Central Fibroblast Growth Factor 21 Browns White Fat via Sympathetic Action in Male Mice. Endocrinology.

[44] Rousset S., Alves-Guerra M.C., Mozo J., Miroux B., Cassard-Doulcier A.M., Bouillaud F., Ricquier D. (2004). The biology of mitochondrial uncoupling proteins. Diabetes.

[45] Van Marken Lichtenbelt W.D., Vanhommerig J.W., Smulders N.M., Drossaerts J.M., Kemerink G.J., Bouvy N.D., Schrauwen P., Teule G.J. (2009). Cold-activated brown adipose tissue in healthy men. N. Engl. J. Med..

[46] Cypess A.M., Lehman S., Williams G., Tal I., Rodman D., Goldfine A.B., Kuo F.C., Palmer E.L., Tseng Y.H., Doria A. (2009). Identification and importance of brown adipose tissue in adult humans. N. Engl. J. Med..

[47] Saito M., Okamatsu-Ogura Y., Matsushita M., Watanabe K., Yoneshiro T., Nio-Kobayashi J., Iwanaga T., Miyagawa M., Kameya T., Nakada K. (2009). High incidence of metabolically active brown adipose tissue in healthy adult humans: Effects of cold exposure and adiposity. Diabetes.

[48] Nagase I., Yoshida T., Kumamoto K., Umekawa T., Sakane N., Nikami H., Kawada T., Saito M. (1996). Expression of uncoupling protein in skeletal muscle and white fat of obese mice treated with thermogenic beta 3-adrenergic agonist. J. Clin. Investig..

[49] Barbatelli G., Murano I., Madsen L., Hao Q., Jimenez M., Kristiansen K., Giacobino J.P., de Matteis R., Cinti S. (2010). The emergence of cold-induced brown adipocytes in mouse white fat depots is determined predominantly by white to brown adipocyte transdifferentiation. Am. J. Physiol. Endocrinol. Metab..

[50] Xue B., Rim J.S., Hogan J.C., Coulter A.A., Koza R.A., Kozak L.P. (2007). Genetic variability affects the development of brown adipocytes in white fat but not in interscapular brown fat. J. Lipid Res..

[51] Cohen P., Levy J.D., Zhang Y., Frontini A., Kolodin D.P., Svensson K.J., Lo J.C., Zeng X., Ye L., Khandekar M.J. (2014). Ablation of PRDM16 and beige adipose causes metabolic dysfunction and a subcutaneous to visceral fat switch. Cell.

[52] Barbera M.J., Schluter A., Pedraza N., Iglesias R., Villarroya F., Giralt M. (2001). Peroxisome proliferator-activated receptor α activates transcription of the brown fat uncoupling protein-1 gene. A link between regulation of the thermogenic and lipid oxidation pathways in the brown fat cell. J. Biol. Chem..

[53] Defour M., Dijk W., Ruppert P., Nascimento E.B.M., Schrauwen P., Kersten S. (2018). The Peroxisome Proliferator-Activated Receptor α is dispensable for cold-induced adipose tissue browning in mice. Mol. Metab..

[54] Owen B.M., Ding X., Morgan D.A., Coate K.C., Bookout A.L., Rahmouni K., Kliewer S.A., Mangelsdorf D.J. (2014). FGF21 acts centrally to induce sympathetic nerve activity, energy expenditure and weight loss. Cell Metab..

[55] Tvrdik P., Asadi A., Kozak L.P., Nedergaard J., Cannon B., Jacobsson A. (1997). Cig30, a mouse member of a novel membrane protein gene family, is involved in the recruitment of brown adipose tissue. J. Biol. Chem..

[56] Westerberg R., Tvrdik P., Unden A.B., Mansson J.E., Norlen L., Jakobsson A., Holleran W.H., Elias P.M., Asadi A., Flodby P. (2004). Role for ELOVL3 and fatty acid chain length in development of hair and skin function. J. Biol. Chem..

[57] Westerberg R., Mansson J.E., Golozoubova V., Shabalina I.G., Backlund E.C., Tvrdik P., Retterstol K., Capecchi M.R., Jacobsson A. (2006). ELOVL3 is an important component for early onset of lipid recruitment in brown adipose tissue. J. Biol. Chem..

[58] Jorgensen J.A., Zadravec D., Jacobsson A. (2007). Norepinephrine and rosiglitazone synergistically induce Elovl3 expression in brown adipocytes. Am. J. Physiol. Endocrinol. Metab..

[59] Nakagawa Y., Shimano H., Yoshikawa T., Ide T., Tamura M., Furusawa M., Yamamoto T., Inoue N., Matsuzaka T., Takahashi A. (2006). TFE3 transcriptionally activates hepatic IRS-2, participates in insulin signaling and ameliorates diabetes. Nat. Med..

